# Supplementary material for: CANT1 Is Involved in Collagen Fibrogenesis in Tendons by Regulating the Synthesis of Dermatan/Chondroitin Sulfate Attached to the Decorin Core Protein
Source: Int J Mol Sci. 2025 Mar 10;26(6):2463. doi: 10.3390/ijms26062463 (PMC11941851; doi:10.3390/ijms26062463)
Supplement: Supplementary file 1 [file ijms-26-02463-s001.zip › Supporting Informatin (revise).pdf]

Supplementary Materials

Article title:

CANT1 Is Involved in Collagen Fibrogenesis in Tendons By Regulating The Synthesis of Dermatan/Chondroitin Sulfate Attached to The Decorin Core Protein

Journal name:

International Journal of Molecular Sciences

Author names:

Rina Yamashita, Saki Tsutsui, Shuji Mizumoto, Takafumi Watanabe, Noritaka Yamamoto, Kenta Nakano, Shuhei Yamada, Tadashi Okamura and Tatsuya Furuichi

Corresponding author:

Tatsuya Furuichi, Ph.D., D.V.M.

Laboratory of Laboratory Animal Science and Medicine, Graduate School of Veterinary Sciences, Iwate University, Japan

E-mail: furuichi@iwate-u.ac.jp

Contents:

1) Table S1–S6
2) Figure S1–S3
3) Information S1

Table S1. Disaccharide composition of chondroitin sulfate/dermatan sulfate in Achilles tendon

Disacccharide unit	Control	*Cant1*$^{-/-}$	*p*-value
	(pmol disaccharide/μg total protein)		
ΔHexUA-GalNAc	44.4 ± 12.8	20.4 ± 2.7	0.01
ΔHexUA-GalNAc(6S)	6.59 ± 1.4	N.D.	–
ΔHexUA-GalNAc(4S)	129 ± 24.1	34.4 ± 7.0	0.00029
ΔHexUA(2S)-GalNAc(6S)	N.D.	N.D.	–
ΔHexUA(2S)-GalNAc(4S)	14.5 ± 2.1	4.58 ± 0.9	0.00014
ΔHexUA-GalNAc(4S,6S)	N.D.	N.D.	–
ΔHexUA(2S)-GalNAc(4S,6S)	N.D.	N.D.	–
Total CS/DS disaccharides	194 ± 32.7	59.4 ± 7.0	0.00019

Data are shown as the means ± standard deviation; n = 4 (Control, *Cant1*$^{-/-}$). Abbreviations: ΔHexUA, 4,5-unsaturated hexuronic acid; GalNAc, *N*-acetyl-D-galactosamine; 2S, 2-*O*-sulfate; 4S, 4-*O*-sulfate; 6S, 6-*O*-sulfate. N.D., not detected.

Table S2. Disaccharide composition of chondroitin sulfate in Achilles tendon

Disacccharide unit	Control	*Cant1*$^{-/-}$	*p*-value
	(pmol disaccharide/μg total protein)		
ΔHexUA-GalNAc	57.7 ± 18.2	29.8 ± 8.4	0.032
ΔHexUA-GalNAc(6S)	4.22 ± 1.1	N.D.	N.D.
ΔHexUA-GalNAc(4S)	22.5 ± 7.5	17.5 ± 7.9	0.40
ΔHexUA(2S)-GalNAc(6S)	N.D.	N.D.	N.D.
ΔHexUA(2S)-GalNAc(4S)	N.D.	N.D.	N.D.
ΔHexUA-GalNAc(4S,6S)	N.D.	N.D.	N.D.
ΔHexUA(2S)-GalNAc(4S,6S)	N.D.	N.D.	N.D.
Total CS disaccharides	84.4 ± 26.0	47.4 ± 13.8	0.045

Data are shown as the means ± standard deviation; n = 4 (Control, *Cant1*$^{-/-}$).
Abbreviations: ΔHexUA, 4,5-unsaturated hexuronic acid; GalNAc, *N*-acetyl-D-galactosamine; 2S, 2-*O*-sulfate; 4S, 4-*O*-sulfate; 6S, 6-*O*-sulfate; N.D., not detected.

Table S3. Disaccharide composition of dermatan sulfate in Achilles tendon

Disacccharide unit	Control	*Cant1*$^{-/-}$	*p*-value
	(pmol disaccharide/μg total protein)		
ΔHexUA-GalNAc	N.D.	N.D.	–
ΔHexUA-GalNAc(6S)	N.D.	N.D.	–
ΔHexUA-GalNAc(4S)	78.4 ± 14.9	7.42 ± 5.4	0.00011
ΔHexUA(2S)-GalNAc(6S)	N.D.	N.D.	–
ΔHexUA(2S)-GalNAc(4S)	15.8 ± 2.8	4.50 ± 1.3	0.00031
ΔHexUA-GalNAc(4S,6S)	N.D.	N.D.	–
ΔHexUA(2S)-GalNAc(4S,6S)	N.D.	N.D.	–
Total DS disaccharides	94.2 ± 17.2	11.9 ± 6.7	0.00011

Data are shown as the means ± standard deviation; n = 4 (Control, *Cant1*$^{-/-}$). Abbreviations: ΔHexUA, 4,5-unsaturated hexuronic acid; 2S, 2-*O*-sulfate; 4S, 46-*O*-sulfate; 6S, 6-*O*-sulfate. N.D., not detected.

Table S4. Disaccharide composition of hyaluronan in Achilles tendon

Disacccharide unit	Control	*Cant1*$^{–/–}$	*p*-value
	(pmol disaccharide/*µ*g total protein)		
ΔHexUA-GlcNAc	13.4 ± 4.1*	7.8 ± 1.7	0.048

Data are shown as the means ± standard deviation; n = 4 (Control, *Cant1*$^{−/−}$). Abbreviations: ΔHexUA, 4,5-unsaturated hexuronic acid; GlcNAc, *N*-acetyl-D-glucosamine.

Table S5. Correspondence between the types of analyses, types of tendon used, and mouse age

Type of analysis	Type of tendon used	Mouse age	Figure
Optical microscopy analysis-1	petellar tendon	P1, P30, P180	Figure 1A–F
Optical microscopy analysis-2	tail tendon	P180	Figure 2A–E
Tensile testing	tail tendon	P210	Figure 2F
Electron microscopy analysis	Achiles tendon	P180	Figure 3A–C
Disaccharide composition analysis	Achiles tendon	P330	Table 1
Western blot analysis	tail tendon	P30, p180	Figure 4A,B
BrdU incorporation analysis	petellar tendon	P4, P10	Figure 5A
Real-time PCR analysis	Achiles tendon	P40	Figure 5B

Table S6. Primer list used in this study

Gene	Forward Primer (5′–3′)	Reverse Primer (5′–3′)
Scx	AAGACGGCGATTCGAAGTTAGAAG	TCTCTCTGTTCATAGGCCCTGCTCATAG
Mkx	GATGGCGACTCCTGCTCTGA	CGGTCTGCCGCCAGCTTTTA
Col1a1	TTGGGGCAAGACAGTCATCGAAT	TTGGGGTGGAGGGAGTTTACACGAA
Tnmd	CTTTACTAGGCTACTACCCATACCCCTACT	ATATATTGGCTAACAGAAGGTTAAGCGTTT
Fmod	GGTGCTTAGACTGTGCTACGG	TCTTCAGAGGCAGGATGGTGATAC
Dcn	ACCGTTATGGAGAATGGCAG	GCGGAGATGTTGTTGTTGTG
Gapdh	ATGTGTCCGTCGTGGATCTG	AGGTGGAAGAGTGGGAGTTG

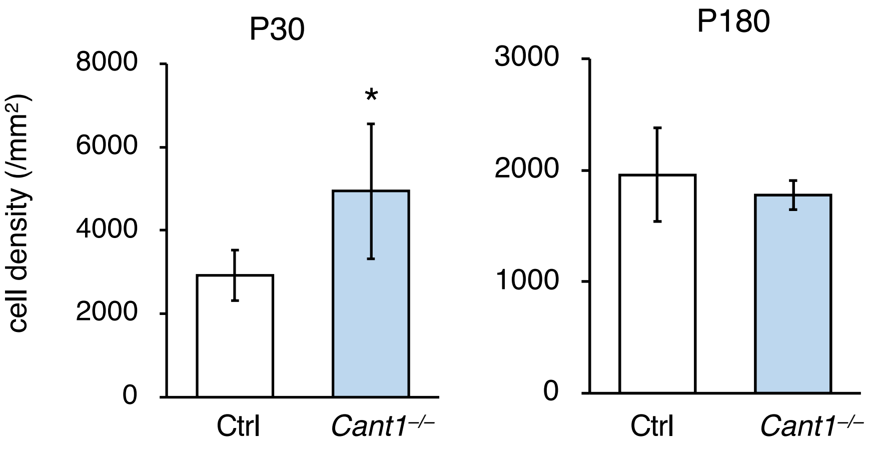

Figure S1. Quantification of cell density in the Achilles tendons of the Ctrl and *Cant1*$^{-/-}$ mice. Cell density was measured using HE-stained sections of the Achilles tendon. Values represent means ± SD (n = 4). * $p < 0.05$ between the Ctrl and *Cant1*$^{-/-}$ mice.

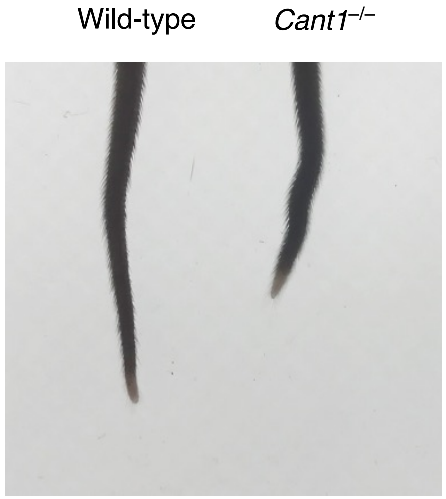

Figure S2. *Cant1*$^{-/-}$ mice exhibited mildly wavy tails at postnatal day 30.

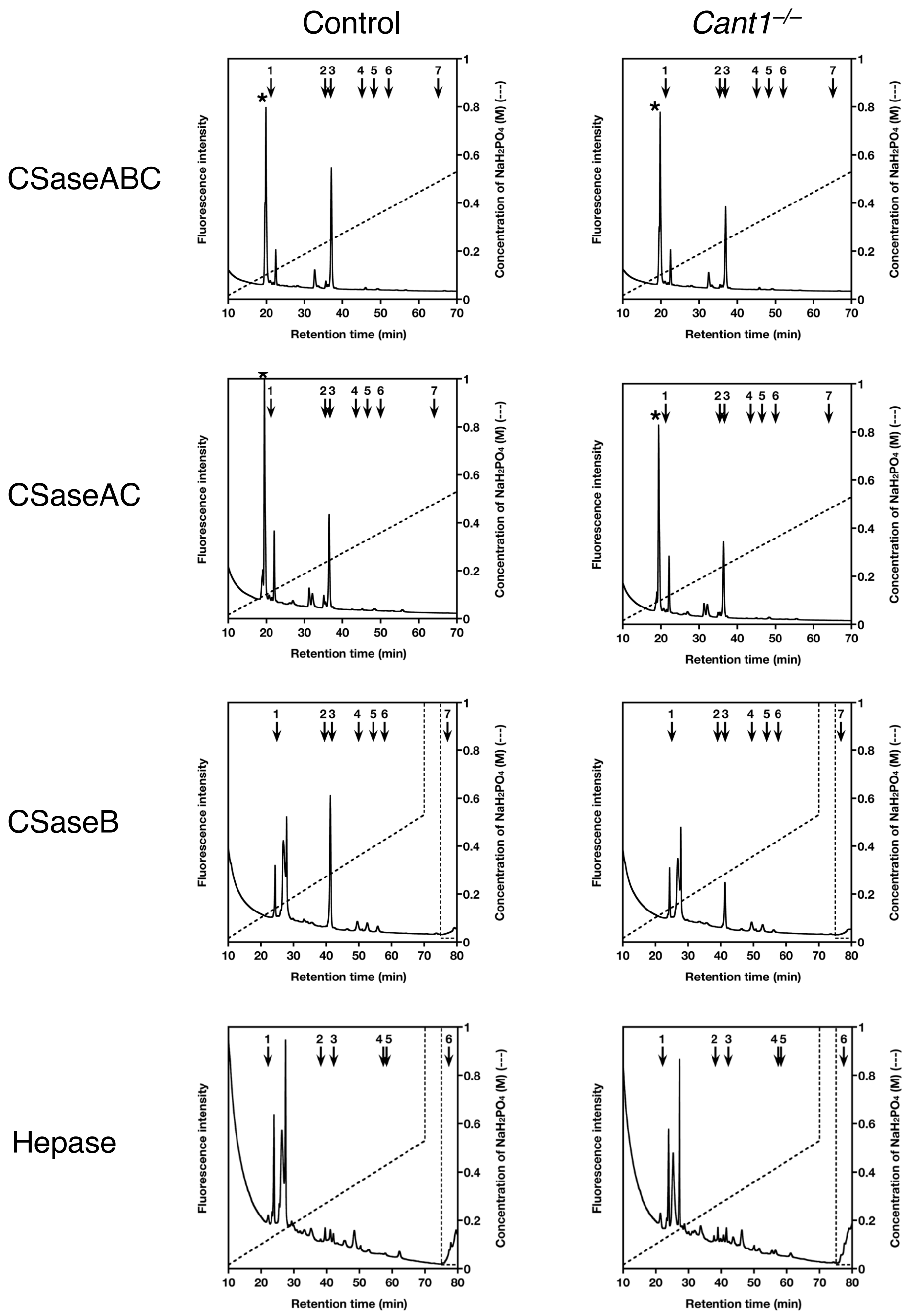

Figure S3. Chromatograms of the CSase or Hepase digests of GAGs derived from the Achilles tendon of the Ctrl and *Cant1*$^{-/-}$ mice. The elution positions of authentic 2AB-labeled CS/DS disaccharides are indicated by numbered arrows in the upper six panels: 1, ΔHexUA-GalNAc; 2, ΔHexUA-GalNAc(6S); 3, ΔHexUA-GalNAc(4S); 4, ΔHexUA(2S)-GalNAc(6S); 5, ΔHexUA(2S)-GalNAc(4S); 6, ΔHexUA-GalNAc(4S,6S); 7, ΔHexUA(2S)-GalNAc(4S,6S). The asterisks indicate ΔHexUA-GlcNAc derived from hyaluronan. The elution positions of authentic 2AB-labeled HS disaccharides are indicated by numbered arrows in the bottom panels: 1, ΔHexUA-GlcNAc; 2,

ΔHexUA-GlcNAc(6S); 3, ΔHexUA-GlcN(NS); 4, ΔHexUA-GlcN(NS,6S); 5, ΔHexUA(2S)-GlcN(NS); 6, ΔHexUA(2S)-GlcN(NS,6S). Abbreviations: ΔHexUA, 4,5-unsaturated hexuronic acid; GalNAc, *N*-acetyl-D-galactosamine; GlcNAc, *N*-acetyl-D-glucosamine; GlcN, D-glucosamine; 2S, 4S, 6S, and NS, 2-*O*-, 4-*O*-, 6-*O*-, and 2-N-sulfate, respectively.

Information S1. The rationality behind the mouse ages used in the respective analyses

Mice of different ages were used in this study: newborn (postnatal day1, P1), infant (P4, P10), juvenile (P30, P40), adult (P180), and middle-aged (P330). To examine the effects of CANT1 deficiency on postnatal tendon development, optical microscopy analysis was performed using mice on P1, P30, and P180. Tensile testing and electron microscopy analyses were performed on adult mice. Disaccharide composition analysis was performed using middle-aged mice. Because it was difficult to prepare mice of several different ages for each of these three studies, they were conducted using mice of the above ages, which were considered reasonable for each analysis. Western blot analysis was performed using juvenile and adult mice to examine decorin production in these stages. BrdU incorporation analysis was performed using infant mice because tendon cell proliferation is active for the first few days of life in mice and then declines significantly. Real-time PCR analysis was performed using juvenile mice because tendon ECM production is active during this stage.
